# Microbiota Induced Changes in the Immune Response in Pregnant Mice

**DOI:** 10.3389/fimmu.2019.02976

**Published:** 2020-01-09

**Authors:** Marijke M. Faas, Yuanrui Liu, Theo Borghuis, Carolien A. van Loo-Bouwman, Hermie Harmsen, Paul de Vos

**Affiliations:** ^1^Immunoendocrinology, Division of Medical Biology, Department of Pathology and Medical Biology, University Medical Center Groningen, University of Groningen, Groningen, Netherlands; ^2^Yili Innovation Center Europe B.V., Wageningen, Netherlands; ^3^Department of Medical Microbiology, University of Groningen, University Medical Center Groningen, Groningen, Netherlands

**Keywords:** pregnancy, gut microbiota, immune response, monocytes, lymphocytes

## Abstract

Pregnancy is associated with adaptations of the immune response and with changes in the gutmicrobiota. We hypothesized the gut microbiota are involved in inducing (part of) the immunological adaptations during pregnancy. To test this hypothesis, we collected feces from pregnant conventional mice before and during pregnancy (days 7, 14, and 18) and microbiota were measured using 16S RNA sequencing. At day 18, mice were sacrificed and splenic (various Th cell populations) and blood immune cells (monocyte subsets) were measured by flow cytometry. The data were compared with splenic and blood immune cell populations from pregnant (day 18) germfree mice and non-pregnant conventional and germfree mice. Finally, the abundances of the individual gut bacteria in the microbiota of each conventional pregnant mouse were correlated to the parameters of the immune response of the same mouse. The microbiota of conventional mice were significantly different at the end of pregnancy (day 18) as compared with pre-pregnancy (Permanova, *p* < 0.05). The Shannon index was decreased and the Firmicutes/Bacteroidetes ratio was increased (Friedman followed by Dunn's test, *p* < 0.05), while abundances of various species (such as *Allobaculum stercoricanis, Barnesiella intestihominis*, and *Roseburia faecis)* were significantly different at day 18 compared with pre-pregnancy. In pregnant conventional mice, the percentage of Th1 cells was decreased, while the percentages of Treg cells and Th2 cells were or tended to be increased vs. non-pregnant mice. In germfree mice, only the percentage of Th1 cells was decreased in pregnant vs. non-pregnant mice, with no effect of pregnancy on Treg and Th2 cells. The percentages of monocyte subsets were affected by pregnancy similarly in conventional and germfree mice. However, the activation status of monocytes (expression of CD80 and MHCII) was affected by pregnancy mainly in conventional mice, and not in germfree mice. Correlation (Spearman's coefficient) of pregnancy affected microbiota with pregnancy affected immune cells, i.e., immune cells that were only affected differently in conventional mice and germfree mice, showed 4 clusters of bacteria and 4 clusters of immune cells, some of these clusters were correlated with each other. For instance, the microbiota in cluster 1 and 2 (in which there were various short chain fatty acid producing microbiota) are positively correlated with immune cells in cluster B, containing Treg cells and Th2 cells. Microbiota and immune cells are affected by pregnancy in mice. The different immunological adaptations to pregnancy between conventional and germfree mice, such as the increase in Treg and tendency to an increase in Th2 cells in conventional pregnant mice only, may suggest that the microbiota may play a role in adapting the maternal immune response to pregnancy.

## Introduction

Pregnancy is characterized by many changes in the immune response in order to tolerate the semi-allogeneic fetus (1). Changes are observed in the peripheral immune response as well as locally at the maternal-fetal interface. At the maternal fetal interface, there is an increased number of uterine NK cells and macrophages (2), which are important for placenta development. Peripherally, the pregnant immune response is amongst others associated with a shift away from a Th1 immune response (3, 4) and increased numbers of regulatory T cells (Treg cells) as well as a decreased number of Th17 cells (5–7). In the periphery, also changes in the innate immune response can be found, such as an increased number monocytes and granulocytes during pregnancy (8, 9). The innate immune response is further characterized by an increased activation monocytes and granulocytes (10, 11) and differences in cytokine production of these cells (9, 12). Moreover, changes in monocyte subsets can be found during pregnancy (13). Similar changes in the immune response have been observed in rodents (7).

The mechanisms that induces these changes in immune responses in pregnancy are only partly understood. Although, the immunological adaptations are necessary in order to tolerate the semiallogeneic fetus, semiallogeneity is only partly responsible for inducing the changes in the maternal immune response, since changes in the immune response do also occur in mice with syngeneic pregnancies (14). Hormonal changes during pregnancy, such as increased progesterone have been shown to be involved in adapting the maternal immune response to pregnancy (15). Physical contact with the placenta during placental circulation may be another mechanism by which innate immune cells are activated during pregnancy (16). However, it has also been shown that the placenta produces many factors into the maternal circulation (17): such as cytokines (18), extracellular vesicles (19), but also fetal DNA (20) may affect immune responses. Other factors suggested to be involved in the adaptation of the immune response to pregnancy are semen (21) or ovarian factors (22).

With the recent knowledge on the role of the gut microbiota in development and maintenance of immune responses (23), we hypothesized that the gut microbiota may be involved in inducing (part of) the immunological changes observed during pregnancy. It has been shown that the gut microbiota may influence the numbers and activation status of various immune cells, such as for instance Treg (24) and Th17 (25) cells. Also, monocyte numbers and their activational state may be influenced by the microbiota (26). As indicated above, these immune cells are also affected by pregnancy. Interestingly, it has been shown that in humans the gut microbiota changes during pregnancy (27), while recently, we have shown that also the mouse gut microbiota changes at the end of pregnancy (14). Moreover, we have shown that the changes in the mouse gut microbiota during pregnancy correlated to differences in expression of immunological changes in the colon (14), suggesting that the gut microbiota affects intestinal immune responses during pregnancy.

In the present study, we aimed in evaluating whether the gut microbiota may also influence the peripheral immune response during pregnancy. Therefore, we first analyzed changes in the gut microbiota during pregnancy in conventional mouse by collecting feces before pregnancy and at days 7, 14, and 18 of pregnancy. We also evaluated the peripheral immune response in these pregnant conventional mice at day 18 of pregnancy. We hypothesized that if some of the peripheral immunological changes would be induced by the microbiota, these changes would not be observed in germfree pregnant mice. Therefore, we also evaluated the peripheral immune response in pregnant germfree mice and compared this to the immune response in the conventional pregnant mice. In the present study we used syngeneic pregnancies, to exclude the effect of semiallogeneity on the immune response to be able to show effects of the microbiota more clearly. Finally, we correlated the gut microbiota to peripheral immunological changes at day 18 of pregnancy in conventional mice.

## Materials and Methods

### Animals

All mice experiment for this study were approved by the Central Committee for Animal experimentation in The Netherlands and experiments were performed according to their guidelines. Male and female wild type conventional C57BL/6JOlaHsd mice were purchased from Envigo (Envigo, Horst, The Netherlands) at an age between 2.5 and 3 months. All conventional mice were cohoused in isolated ventilated cages (5 mice per cage) with a 12-h light,12-h dark cycle. Germfree mice (C57BL/6JOlaHsd) were bred and nurtured at the Central Animal Facility of the UMCG. They were kept in germfree isolators until sacrifice and checked for germfree status regularly by the facility. Germfree mice were cohoused (5 mice per cage) with a 12-h light,12-h dark cycle. All (conventional and germfree) mice were provided with germfree bedding, an irradiated diet, AIN-93M (Research Diet Services, the Netherlands) and sterile water *ad-libitum*. The diet was provided starting at least 3 weeks before pregnancy and continuing during pregnancy and 3 weeks before sacrifice in non-pregnant mice.

After 3-weeks acclimatization in the facility and on the diet, vaginal smears were taken to check the ovarian cycle of the mice. When in pro-estrus, the female mice were placed with male mice overnight. We used syngeneic pregnancies, to exclude the effect of semiallogeneity on the immune response. The next morning, mice were checked for a plug or sperm in the smear and this day is day 0 of pregnancy. For conventional mice, fecal samples were collected before and during pregnancy (on days 7, 14, and 18; *n* = 12). These conventional mice were terminated at day 18 of pregnancy. Germfree pregnant mice (*n* = 9) were also terminated at day 18. Two groups of non-pregnant mice [1 group of conventional (*n* = 12) and 1 group of germfree mice (*n* = 11)] were terminated for measuring the immune response at di-estrus of the ovarian cycle to ensure low levels of estrogen and progesterone. Termination was done in sterile flow cabinets. At termination, mice were anesthetized with isofluorane/O_2_, and blood was collected from the aorta into EDTA tubes (BD-Plymouth, UK). After bleeding, spleens were also collected. For pregnant mice, we counted number of viable fetuses and number of resorptions and weighed individual placentas and fetuses.

### Microbiota Measurement

Immediately after collection, fecal samples were snap frozen in screw caps in liquid nitrogen and stored at −80°C until measurement. For DNA isolation 0.25 gr of fecal sample was added to a 2 ml sterile bead beater tube filled with 1 ml of 0.1 mm zirconia/silica beads with 1 ml of lysis buffer (5 M NaCl, 1 M Tris-HCL (pH = 8); 0.5 M EDTA and 10 % SDS). Samples were placed in a bead beater (Percellys 24, Bertin Instruments, Montigny-leBretonneux, France) for 3 min on 5,500 RPM in three cycles of 30–60 s. The samples were heated to 95°C for 15 min while shaking every 5 min and put on ice for 5 min. Hereafter, the tubes were centrifuged for 5 min at maximum speed to pellet debris. The supernatant was transferred into a new sterile 2 ml tube and 300 μl of fresh lysis buffer was added followed by bead beating, heating to 95°C and centrifuged for 5 min at maximum speed to remove any additional debris.

Nucleic acids were cleaned from proteins and cell debris by precipitation using ammonium acetate (260 μl of 10 M ammonium acetate) on ice for 5 min. Tubes were centrifuged for 10 min at full speed at 4°C. Pellet was discarded and supernatant was treated a second time with the same procedure, followed by precipitation of the DNA from the supernatant with isopropanol on ice for 30 min. After centrifugation, the pellets were washed with ethanol 70% and dried. Hereafter, genomic DNA purification was performed according to the protocol of the QIAmp DNA Mini Kit (Qiagen, Benelux, Venlo, the Netherlands). DNA concentration was measured with a NanoDrop ND-1000 Spectrophotometer (Thermo Fisher Scientific, Waltham, MA, USA).

### 16S rRNA Gene Sequencing, Quality Control, and Taxonomy Assignment

Subsequently, the DNA was used for the amplification of the V3–V4 region of the 16S rRNA gene using modified 341F (AATGATACGGCGACCACCGAGATCT-ACACTCTTTCCCTACACGACGCTCTTCCGATCT-NNNNCCTACGGGAGGCAGCAG) and 806R primers (CAAGCAGAAGACGGCATACGAGAT-barcode-GTGACTGGAGTTCAGACGTGTGCTCTTCCGATCT-GGACTACHVGGGTWTCTAAT) containing a 6-nucleotide barcode and flow-cell adaptor on the 806R primer as described elsewhere (28). A 2 × 300 cartridge (Illumina, Eindhoven, the Netherlands) was used to perform both MiSeq library preparation and sequencing. PCR protocol, DNA cleanup and the library preparation were all done as described before (29). Sequence reads with a quality score lower than 0.9 were discarded by PANDAseq to increase the quality. QIIME was used to identify sequence reads until the genus levels, while ARB software (29) was used to identify sequences at the species level.

### Isolation and Staining of Spleen Cells

Cells were isolated from the maternal spleen for immune cell staining according to methods described before (14). The spleen was first cut into small pieces, mechanically disrupted between two microscopy slides in 3 ml ice-cold RPMI containing 10% (v/v) heat-inactivated fetal calf serum (FCS). Splenic red blood cells were eliminated by incubation with 4 ml ice-cold ammonium chloride. Falcon tubes with cell strainer caps (Corning, Amsterdam, the Netherlands) (35 μm) were used to remove cell clumps before the cells were counted and used for staining.

All antibodies were diluted and supplemented to a volume of 25 μl with FACS buffer [PBS + 10% FCS (*v/v*)]. Approximately 1 × 10^6^ spleen cells were incubated in Zombie NIR for 30 min. After washing with FACS buffer (2 times), the supernatant was discarded and the cells were incubated for 20 min in FACS buffer [10% FCS (*v/v*)] containing 20% (*v/v*) normal rat serum (Jackson, Newmarket, UK) on ice and in the dark, to prevent non-specific antibody binding. This was followed by incubation in an extracellular antibody mix for 30 min on ice and in the dark (Table 1). After 2 washing steps, the cells were fixed in FACS lysing solution (BD Biosciences, Breda, the Netherlands) for 30 min on ice and in the dark. Then cells were washed twice with permeabilization buffer (eBioscience, Vienna, Austria) after which they were incubated with an intracellular blocking medium (20% (v/v) rat serum in permeabilization buffer) for 20 min. After washing, the cells were incubated with the intracellular antibody mix for 30 min on ice and in the dark (Table 1). This was followed by 2 washing steps with permeabilization buffer. Finally, the cells were taken up in 200 μl ice-cold 2%(v/v) FACS buffer and stored at 4°C until analysis within 24 hrs. FMO controls were used to set the gates.

**Table 1:** Antibody mix for staining of splenic cells.

**Marker**	**Fluorchrome**	**Dilution**	**Supplier**	**Mix**
CD4	PerCp-Cy5.5	75x	Biolegend	Extracellular
CD3	BV605	25x	Biolegend	Extracellular
Tbet	BV421	10x	Biolegend	Intracellular
RORyT	PE	100x	eBioscience	Intracellular
Gata3	AF647	100x	BD Pharmingen	Intracellular
FoxP3	Fitc	50x	eBioscience	Intracellular
Dead/live	Zombie NIR	1000x	Biolegend	

### Staining of Blood Monocytes

Maternal blood was stained for monocytes subsets and activation status according to an adapted method previously used for rat studies (13). Antibody specifications are shown in Table 2. Two hundred microliters of whole blood was diluted with 200 μl RPMI buffer (500 ml RPMI + 50 ml decomplemented FCS), and incubated with 50 μl ice-cold extracellular blocking medium (2% (v/v) Fc-block (purified anti-mouse CD16/32) (Biolegend, San Diego, USA) in 10% (v/v) FACS+EDTA buffer) for 10 min on ice in the dark. After centrifugation (1800 RPM, 5 min, 4°C) the supernatant was discarded and the cells were incubated in 25 μl ice-cold antibody mix for 30 min on ice in the dark. After incubation, the cells were incubated with 1 ml FACS lysing buffer for 15 min at room temperature in the dark. After washing for 3 times, the cells were taken up in 500 μl ice-cold 10% (v/v) FACS+EDTA buffer and stored at 4°C until analysis within 24 hrs.

**Table 2:** Antibody mix for staining of blood monocytes.

**Marker**	**Fluorochrome**	**Dilution**	**Supplier**
MHC2	PerCp-Cy5.5	200x	Biolegend
Ly6C	AF488	200x	Biolegend
CD43	APC	100x	Biolegend
CD11b	PE	50x	Biolegend
CD80	PB	25x	Biolegend
Ly6G	BV605	25x	BD Horizon

### Flow Cytometry

Samples were analyzed by the FACSverse flow cytometer system (BD Biosciences Franklin Lakes, USA), using the FACSsuite software. Analysis was performed by FlowJo version 10 software (FlowJo, LLC, Oregon, USA). Gating strategy was performed as described in **Figures 6A**, **7A**.

### Statistics

For the Shannon index, the PCA, the Permanova and the similarity percentage breakdown (Simper) test we used Past3 (30). For statistical analysis (Graphpad Prism) of differences in Shannon index, Firmicutes/Bacteroidetes ratio, bacterial phyla and species between pre-pregnancy and the days of pregnancy, we used the Friedman's test followed by Dunn's post-test. Data were considered significantly different when *p* < 0.05 and considered a trend if p = between 0.05 and 0.1.

Differences in fetal and placental weight and number of (live) fetuses between pregnant conventional and germfree mice were tested using the Mann-Whitney-U test, data were considered significantly different if *p* < 0.05.

For testing the effect of pregnancy (pregnant vs. non-pregnant mice) or the effect of the germfree status (conventional vs. germfree mice) on immune cells, we used the Two-Way ANOVA (TWA). For this, data were first tested for normality using the Kolmogorov-Smirnov test. Data that were not normally distributed were log transformed before the analysis. Post-testing was performed with Fisher's LSD post-test. Since we were mainly interested in the effect of pregnancy, post-tests were only done on the difference between pregnant and non-pregnant mice in both conventional and germfree mice. Data were considered significantly different when *p* < 0.05.

To gain insight into the relationship between the gut microbiota and immune cell changes during pregnancy, we correlated individual microbiota abundances with immune cell data of the same mice. We correlated individual microbiota that were changed during pregnancy to individual immune cells that were affected by pregnancy only in conventional mice using the spearman's correlation coefficient. The correlation coefficients were visualized in clustered heatmaps using Clustvis (31). Cluster analysis using Euclidian distance and Ward's clustering method was performed.

## Results

**Figure 1:**
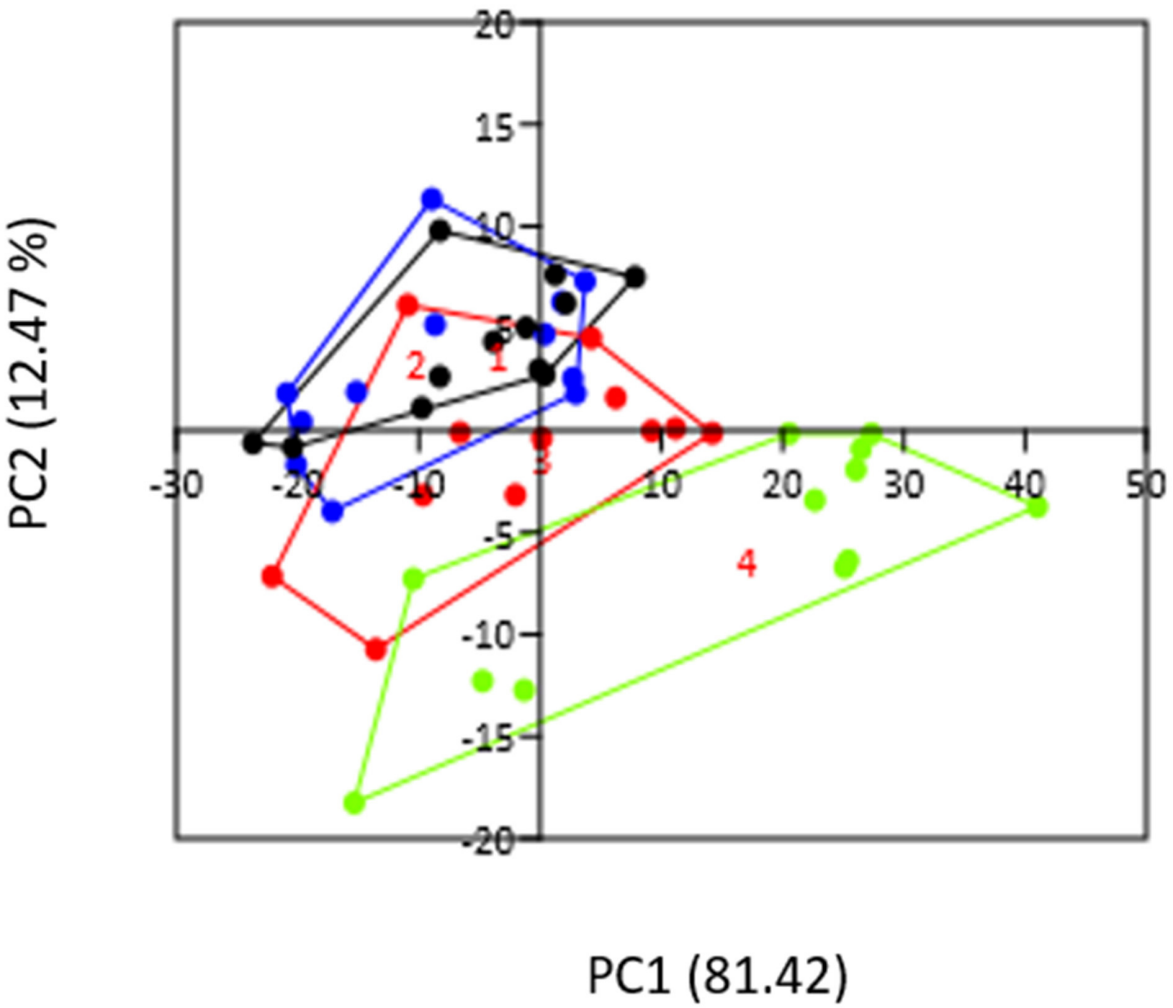
PCA plot representing the gut microbiota of conventional pre-pregnant (black circles), day 7 (blue circles), day 14 (red circles), and day 18 (green circles) mice. The day of pregnancy (PC2) does explain part of the variation. PC1 explains 81.42% of the variation, while PC 2 explains 12.47% of the variation.

We determined the gut microbiota composition of mice after longitudinal sampling of feces from 12 conventional mice starting before pregnancy and at days 7, 14, and 18 of pregnancy. PCA analysis (Figure 1) showed that PC1 explained about 80% of the variation and while PC2, mainly represented by day of pregnancy, explains about 12 % of the variation in our groups. It can be seen that day 18 of pregnancy is significantly different from all other groups (Permanova, *p* < 0.05). Although day 14 of pregnancy also appeared different from pre-pregnancy and from day 7 in the PCA plot, this difference was not statistically significant (Permanova, *p* > 0.05).

To identify bacteria that explained the variation between the pre-pregnancy microbiota and day 18 microbiota, we performed a Simper test (Table 3). We found that *Allobaculum stercoricanis*, which significantly increased at day 18 vs. pre-pregnancy, explained 29 % of the variation between pre-pregnancy and day 18 of pregnancy. *Barnesiella intestinihominis*, which significantly decreased during pregnancy, explained 17% of the variation between the pre-pregnancy and day 18 microbiota. Various bacterial species (such as *Porphyromonas pogonae, Barnesiella viscericola, Clostridium leptum*) explain between 1 and 3% of the variation between the pre-pregnancy and day 18 microbiota.

**Table 3:** The microbiota species explaining the difference in gut microbiome at day 18 of pregnancy vs. pre-pregnancy.

**Species**	**% contribution to day 18 variation**	**Cumulative contribution**	**Mean abundance pre-pregnancy**	**Mean abundance pregnancy day 18**	**Statistical significance**
*Allobaculum stercoricanis*	29.01	29.01	35.1	52.8	*p*<0.05
*Barnesiella intestinihominis*	17.22	46.23	18.4	4.79	*p*<0.05
*Porphyromonas pogonae*	3.908	50.13	6.03	3.19	*p*<0.05
*Barnesiella viscericola*	3.42	53.55	4.27	1.56	*p*<0.05
*Clostridium leptum*	3.295	56.85	1.87	2.95	*ns*
*Faecalitalea cylindroides*	1.79	58.64	2.7	3.85	*p*<0.05
*Olsenella profusa*	1.728	60.37	2.33	1.27	*p*<0.05
*Lactobacillus johnsonii*	1.583	61.95	1.3	0.841	*ns*
*Clostridium papyrosolvens*	1.513	63.46	1.45	0.298	*p*<0.05
*Acetatifactor muris*	1.486	64.95	0.975	1.05	*ns*
*Flavonifractor plautii*	1.444	66.39	0.612	1.29	*ns*
*Clostridium fusiformis*	1.375	67.77	0.603	1.34	*ns*
*Parasutterella excrementihominis*	1.309	69.08	1.74	1.01	*p*<0.05
*Blautia coccoides*	1.293	70.37	0.594	1.09	*ns*
*Romboutsia ilealis*	1.245	71.62	0.537	1.18	*ns*
*Eisenbergiella tayi*	1.245	72.86	0.505	1.2	*ns*
*Alloprevotella rava*	0.9948	73.86	0.957	0.198	*p*<0.05
*Sutterella parvirubra*	0.9198	74.78	1.21	0.689	*p*<0.05
*Mucispirillum schaedleri*	0.8793	75.65	0.142	0.706	*ns*
*Bifidobacterium animalis*	0.8583	76.51	1.16	1.14	*ns*
*Alistipes finegoldii*	0.8434	77.36	0.922	0.85	*ns*
*Bifidobacterium pseudolongum*	0.7753	78.13	0.81	0.515	*ns*
*Olsenella umbonata*	0.7728	78.9	0.897	0.414	*ns*
*Desulfovibrio desulfuricans*	0.7725	79.68	0.552	0.656	*ns*
*Butyrivibrio crossotus*	0.6543	80.33	0.298	0.287	*ns*
*Bacteroides vulgatus*	0.5959	80.93	0.481	0.0983	*ns*
*Roseburia faecis*	0.5823	81.51	0.229	0.528	*p*<0.05
*Anaerotruncus colihominis*	0.5782	82.09	0.279	0.513	*ns*
*Clostridium jejuense*	0.5744	82.66	0.371	0.392	*ns*
*Alistipes senegalensis*	0.545	83.21	0.562	0.678	*ns*
*Natronoflexus pectinivorans*	0.533	83.74	0.518	0.409	*ns*

*Significance (last column) was tested using Friedman's tests on all pregnancy days followed by Dunn's posttest comparing pre-pregnancy with day 18*.

**Figure 2:**
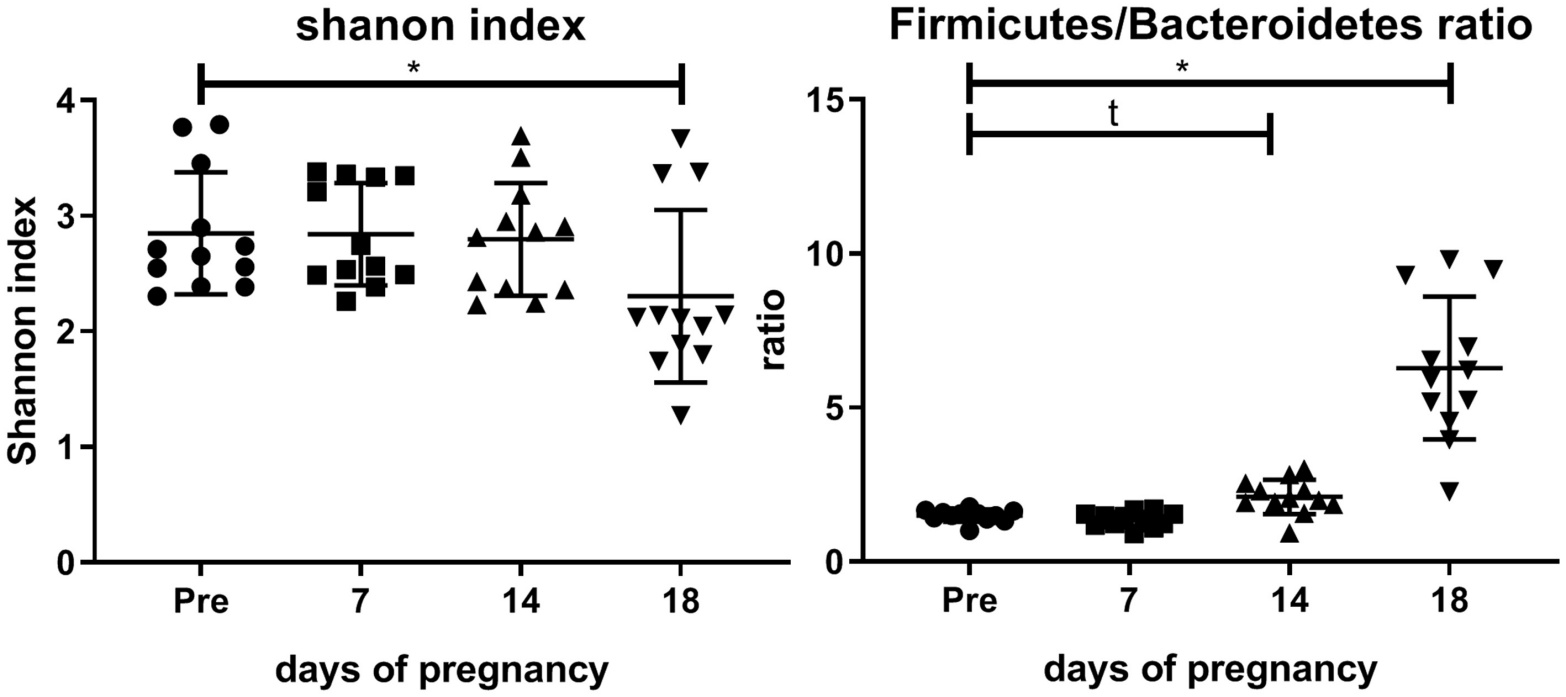
Shannon index (left graph) and Firmicutes/Bacteroidetes ratio (right graph) in the feces of conventional mice measured pre-pregnancy (pre) and at days 7, 14, and 18 of pregnancy (*n* = 10 at each day). ^*^Significantly different from pre-pregnancy (Friedman's test, followed by Dunn's post-test, *p* < 0.05). t, significant trend vs. pre-pregnancy (Friedman's test, followed by Dunn's post-test, *p* < 0.1).

Figure 2 shows the Shannon index and the Firmicutes/Bacteroidetes ratio before and during pregnancy. It can be seen that the Shannon index is significantly decreased at day 18 as compared with pre-pregnancy (Friedman test followed by Dunn's posttest). The Firmicutes/Bacteroidetes ratio is significantly increased at day 18 as compared with pre-pregnancy (Friedman's test followed by Dunn's post-test, *p* < 0.05), while a trend toward an increase of the Firmicutes/Bacteroidetes ratio was seen at day 14 of pregnancy (Friedman test followed by Dunn's posttest, *p* = 0.08).

**Figure 3:**
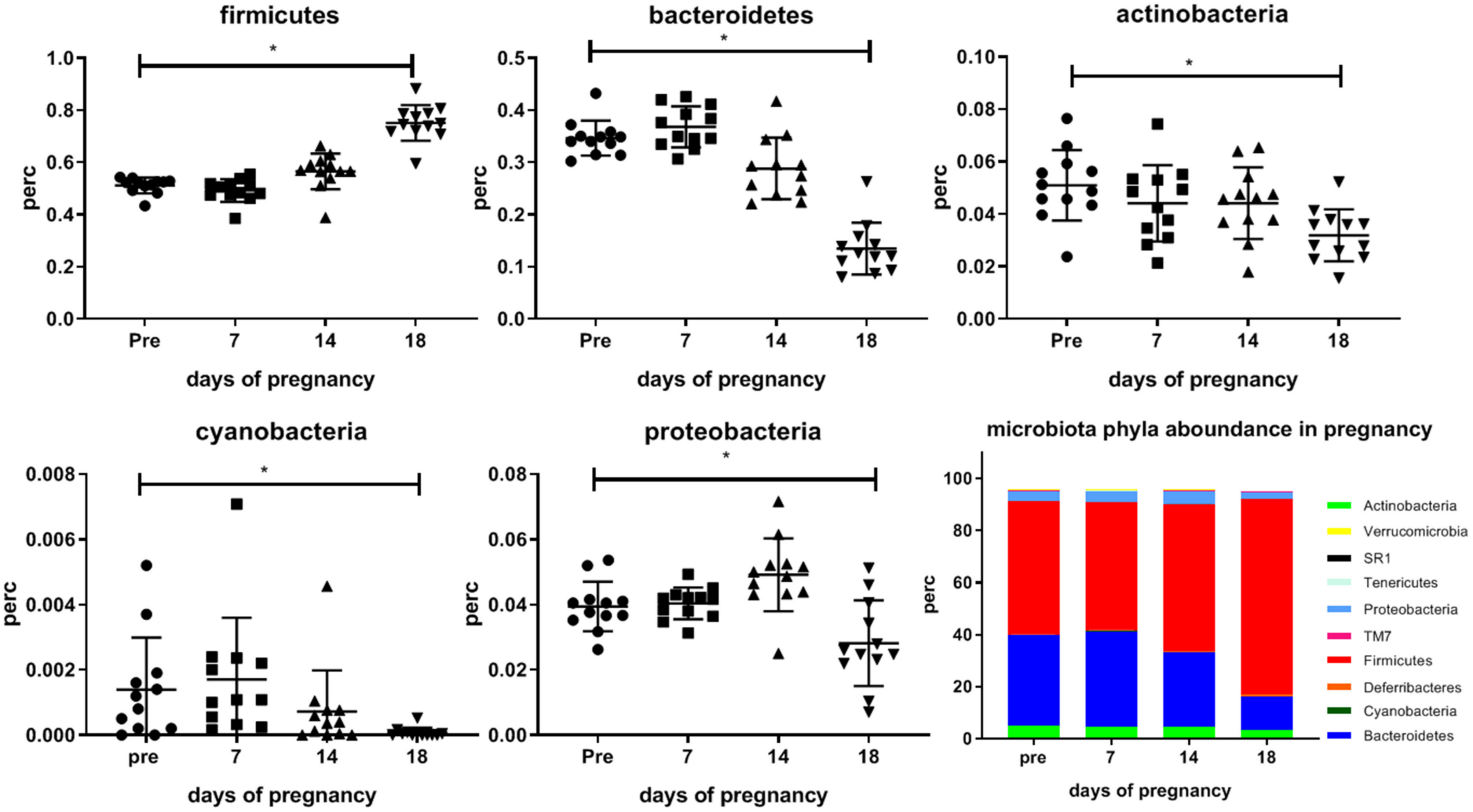
Abundance of the bacterial phyla (Firmicutes, Bacteriodetes, Actinobacteria, Cyanobacteria, and Proteobacteria which significantly differ during pregnancy as compared with pre-pregnancy in the feces of conventional mice measured pre-pregnancy (pre) and during pregnancy at days 7, 14, and 18. Bottom right graph shows stack bars for pre-pregnancy and the 3 days of pregnancy, showing the percentage abundance of the all bacterial phyla present in the feces of these mice. (12 conventional mice were longitudinally studied from pre-pregnancy until the end of pregnancy). ^*^Significantly different from pre-pregnancy (Friedman's test, followed by Dunn's post-test, *p* < 0.05).

Figure 3 shows differences in abundances of various bacterial phyla occurring during pregnancy in the mouse. Significant changes only occurred at day 18 vs. pre-pregnancy. We found a significantly increased abundance of Firmicutes at day 18 as compared with pre-pregnancy (Friedman's test followed by Dunn's posttest, *p* < 0.05), and a significantly decreased abundance of Bacteroidetes, Actinobacteria, Cyanobacteria and Proteobacteria at day 18 of pregnancy as compared with pre-pregnancy (Friedman's test followed by Dunn's post-test, *p* < 0.05).

**Figure 4:**
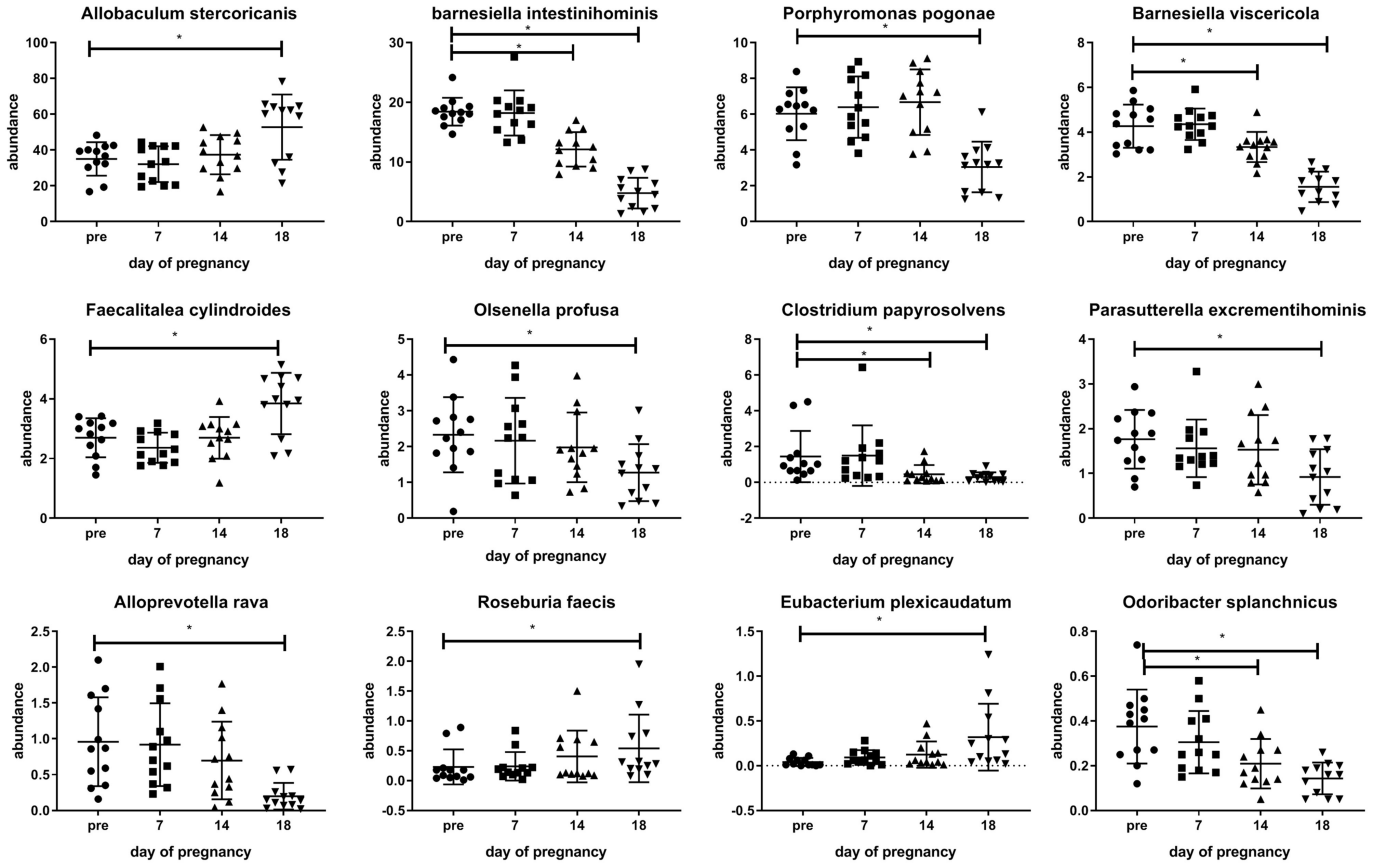
Changes observed in the abundance of various microbiota species in the feces of conventional mice measured pre-pregnancy (pre) and during pregnancy at days 7, 14, and 18. ^*^Significantly different from pre-pregnancy (Friedman's test, followed by Dunn's post-test, *p* < 0.05).

Various changes in the microbiota can also be observed at the species level. Examples of changes at the species level can be seen in Figure 4. In this figure, examples of bacterial species showing differences at day 18 of pregnancy as compared with pre-pregnancy (Friedman's test followed by Dunn's posttest, *p* < 0.05) are shown. Various species of the Firmicutes phylum are increased at day 18 of pregnancy vs. pre-pregnancy, such as *Allobaculum sternicoricanis, Faecalitalea cylindroides, Roseburia faecis, Eubacteria plexicaudatum*, while various other bacterial species, such as various Bacteroidetes species are decreased at day 18 of pregnancy vs. pre-pregnancy (*Barnesiella intestinihominis, B viscericola, Alloprevotella rava, and Odoribacter splanchnicus)*. Some bacteria (*B. intestinihominis, B. viscericola, C. papysosolvens, and O. splanchnicus*) already show significant changes at day 14 of pregnancy as compared with pre-pregnancy (Friedman's test followed by Dunn's post-test, *p* < 0.05). A complete list of all changes can be found in Supplementary Table 1.

### Changes in Immune Cells on Pregnancy Day 18 in Conventional and Germfree Mice

To study the effect of the microbiota on immunological changes during pregnancy in mice, we sacrificed conventional mice on day 18 and collected spleens and blood in order to evaluate lymphocyte subsets in the spleen and monocyte subsets in blood. We hypothesized that if the gut microbiota would be involved in the adaptations of the immune response to pregnancy, some of the adaptations observed in conventional pregnant mice would not be observed in germfree mice.

### Fetal and Placental Weight in Conventional and Germfree Pregnant Mice

**Figure 5:**
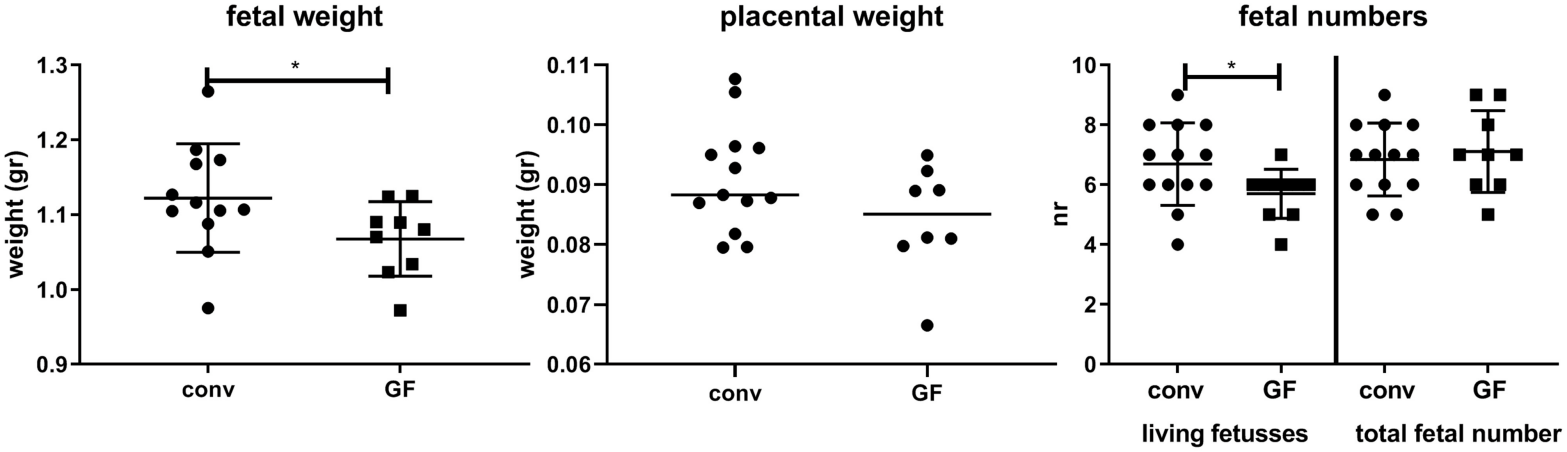
Fetal and placental weight and fetal numbers (living fetuses and total numbers) for conventional day 18 pregnant (conv) and germfree day 18 pregnant (GF) mice. ^*^Significantly different from conventional mice (Mann-Whitney-*U*-test, *p* < 0.05).

Figure 5 shows that fetal weight is slightly, but significantly decreased in germfree mice, with no significant effect on placental weight. Although the total number of fetuses was similar in conventional and germfree mice, the number of resorptions was increased in germfree mice, resulting in a significantly decreased number of live fetuses in germfree mice.

### The Effect of Pregnancy on Thelper Subsets

**Figure 6:**
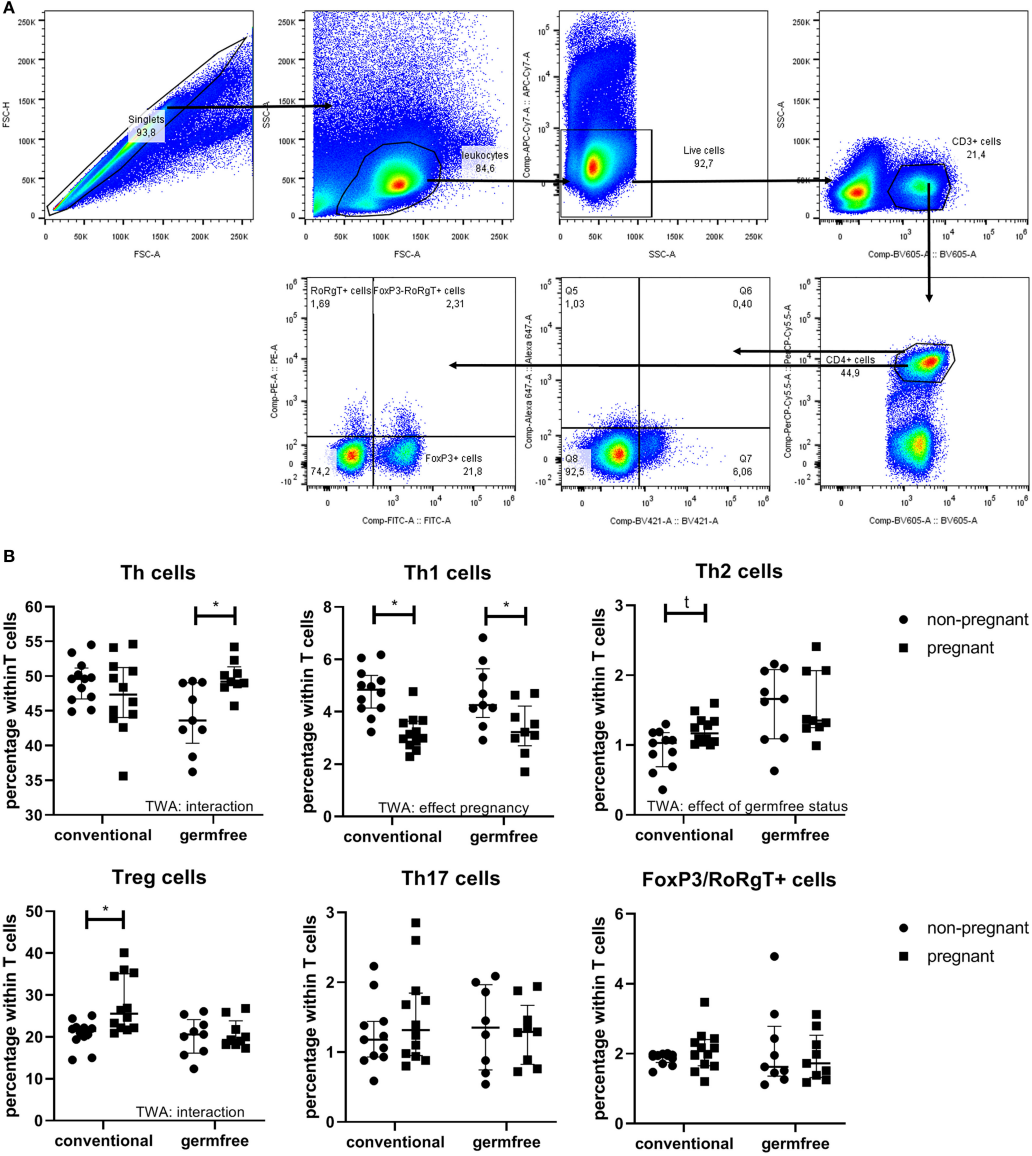
**(A)** Gating strategy for lymphocyte subsets. First singlets and live cells were selected and copied to the FSC-SSC plot. In this plot, leukocytes were selected and copied to an SSC-BV605 plot, in which the CD3+ cells were selected. The CD3+ cells were copied to a BV605-PerCP-Cy5.5 plot to selected the CD4+ cells. CD4 positive cells were copied to a BV421-Alexa647 plot (to select Tbet+ cells and Gata3+ cells) and to a FITC-PE plot (to select FoxP3+ cells and RoRgT+ cells). For gate setting of the latter two plots we used FMOs. **(B)** Percentage of splenic Th cells, Th1 cells, Th2 cells, Treg cells, Th17 cells, and FOXP3/RORgT double positive cells (within the T cell population) of pregnant and non-pregnant conventional and germfree mice. TWA, Two-way ANOVA. ^*^Significantly different from non-pregnant mice (TWA followed by Fisher's LSD post-test, *p* < 0.05). t: significant trend from non-pregnant mice (TWA followed by Fisher's LSD post-test, *p* < 0.1).

Figure 6B shows the percentage of Thelper (Th) cells, T-box transcription factor+ (Tbet) cells (Th1 cells), Gata binding protein 3+ (Gata3) cells (Th2 cells), Forkhead box P3+ (FoxP3) cells (Treg cells), RAR-related orphan receptor gamma t+ (RoRgT) cells (Th17 cells) and FoxP3/RoRgT double positive cells. For Th cells, the Two-Way ANOVA (*p* < 0.05) indicated that there was interaction between pregnancy and the germfree status, indicating that the effect of pregnancy on Th cells was different in conventional compared with germfree mice. Post-testing showed an increased percentage of Th cells in pregnant germfree mice vs. non-pregnant germfree mice, but no difference in Th cells in pregnant vs. non-pregnant conventional mice. The percentage of Tbet+ cells is decreased by pregnancy (TWA, *p* < 0.05), but not affected by the germfree status (TWA, *p* < 0.05). Post testing showed that in both conventional and germfree pregnant mice the percentage of Tbet+ cells is decreased as compared with the percentage of Tbet+ cells in non-pregnant mice. The percentage of GATA3+ cells is increased in pregnant conventional mice as compared with non-pregnant conventional mice, but no difference in percentage of GATA3+ cell was found between pregnant and non-pregnant germfree mice (TWA followed by Fisher LSD test, *p* < 0.05). TWA also showed an effect of the germfree status, i.e., an increase of GATA3+ cells in germfree mice.

FoxP3+ cells were affected by both pregnancy (increase) and the germfree status (decrease). (TWA, *p* < 0.05). Post testing revealed a significant increase in percentage FoxP3+ cells in conventional pregnant mice vs. conventional non-pregnant mice, but not in pregnant vs. non-pregnant germfree mice. No effect of pregnancy or the germfree status was observed on RoRgT+ or on FoxP3/RoRgT double positive cells.

### Effect of Pregnancy on Leucocyte Subsets in Blood

**Figure 7:**
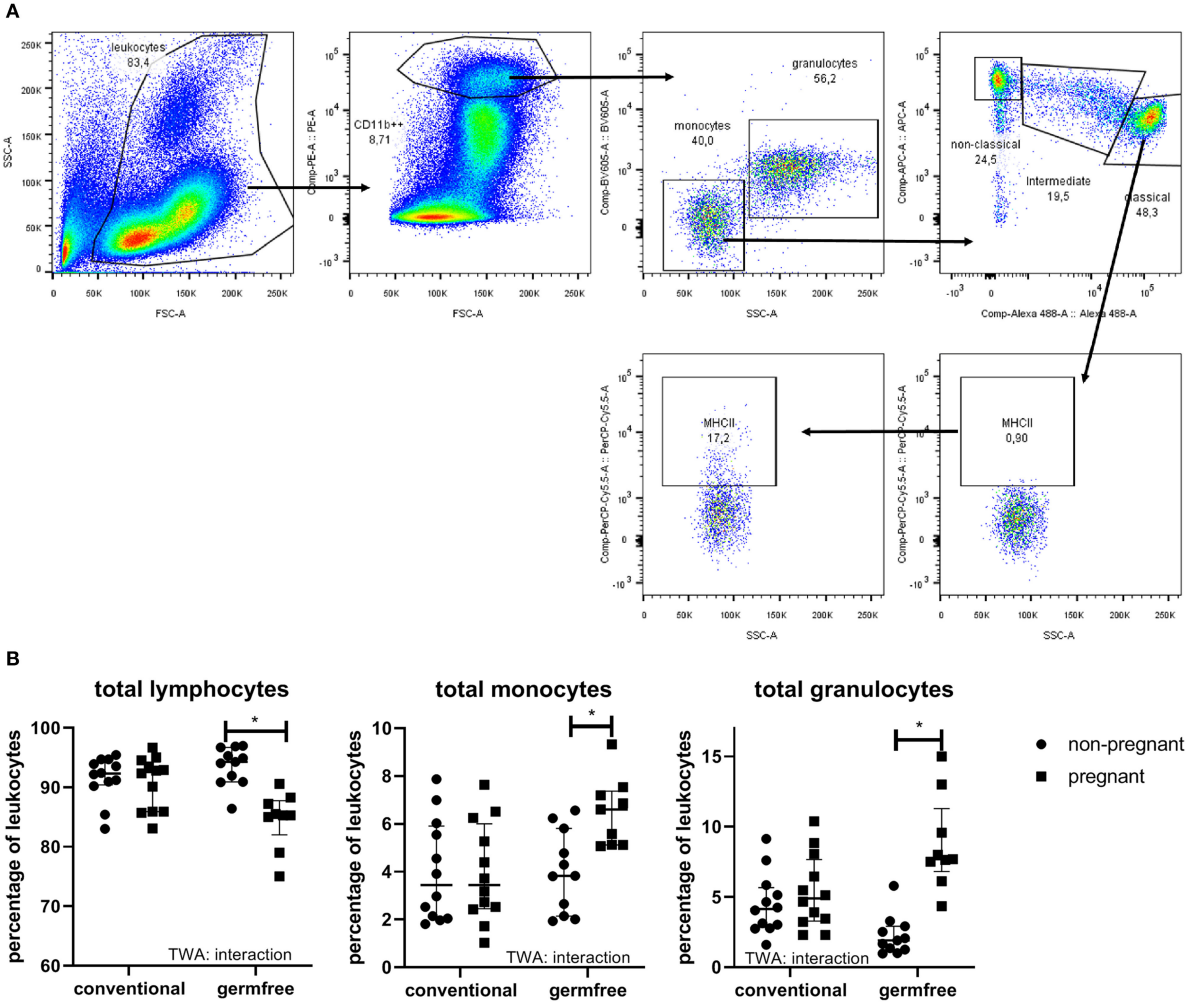
**(A)** Gating strategy for leukocyte populations and monocytes. In the FSC-SSC plot, we selected the leukocytes, which were copied to the FSC-PE plot to select CD11b++ cells (myeloid cells). These myeloid cells were copied to an SSC-BV605 plot to discriminate monocytes from granulocytes. Percentages of monocytes and granulocytes were derived from this plot. Percentage lymphocytes was 100% of myeloid cells. The monocytes were then copied to an Alexa488-APC plot to discriminate classical, intermediate and non-classical monocytes. Each of these three subsets were then copied to an SSC-PerCPCy5.5, to select MHCII+ cells (example is shown for classical monocytes), using the FMO to set the gates. Each of these monocytes' subsets were also copied to an SSC-Pacific Blue plot to select the CD80+ cells, using FMO to set the gates, similar to MHCII (not shown in the figure). **(B)** Percentage of lymphocytes, monocytes, and granulocytes in whole blood of pregnant (day 18) and non-pregnant conventional and germfree mice. TWA, Two-way ANOVA. ^*^Significantly different from non-pregnant mice (TWA followed by Fisher's LSD post-test, *p* < 0.05).

The different leukocyte subsets in the blood are shown in Figure 7B. TWA showed an interaction between pregnancy and the germfree status for lymphocytes. Post-testing showed a significantly decreased percentage of lymphocytes in germfree pregnant mice as compared with germfree non-pregnant mice, but no difference in percentage of lymphocytes in pregnant vs. non-pregnant conventional mice (TWA followed by Fisher LSD test, *p* < 0.05). Also, for monocytes and granulocytes, we found an interaction between pregnancy and germfree status (TWA, *p* < 0.05). Only in germfree mice (Fisher LSD test, *p* < 0.05), not in conventional mice, we found a significantly increased percentage of monocytes and granulocytes in pregnant vs. non-pregnant mice.

### The Effect of Pregnancy on Monocyte Subsets

**Figure 8:**
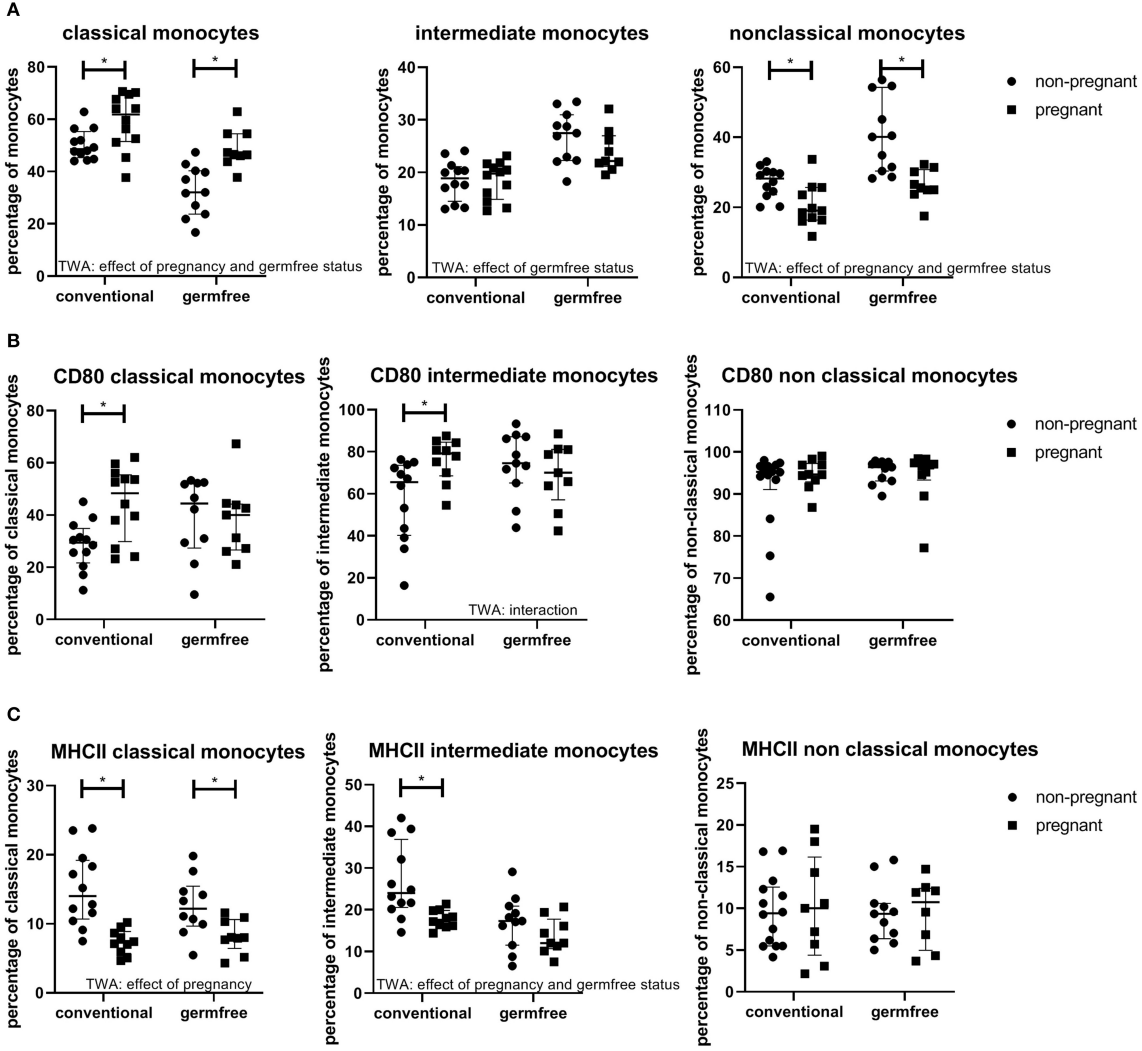
**(A)** Percentages of monocyte subsets (within the monocyte population) in blood of conventional and germfree pregnant and non-pregnant mice. TWA, Two-way ANOVA. ^*^Significantly different from non-pregnant mice (TWA followed by Fisher's LSD post-test, *p* < 0.05). **(B)** Percentage CD80 positive classical, intermediate and non-classical monocytes in pregnant and non-pregnant conventional and germfree mice. TWA, Two-way ANOVA. ^*^Significantly different from non-pregnant mice (TWA followed by Fisher's LSD post-test, *p* < 0.05). **(C)** Percentage MHCII positive classical, intermediate and non-classical monocytes in pregnant and non-pregnant conventional and germfree mice. TWA, Two-way ANOVA. ^*^Significantly different from non-pregnant mice (TWA followed by Fisher's LSD post-test, *p* < 0.05).

It can be observed from Figure 8A that the percentage of classical monocytes was affected by pregnancy (TWA, *p* < 0.05); the percentage of classical monocytes is increased in pregnant mice as compared with non-pregnant mice, both in conventional and germfree mice (Fisher LSD test, *p* < 0.05). Germfree status also affected the classical monocytes, i.e., classical monocytes are decreased in germfree mice (TWA, *p* < 0.05) as compared with conventional mice. There was no effect of pregnancy on the percentage of intermediate monocytes, while there was an effect of germfree status, i.e., in germfree mice the percentage of intermediate monocytes was increased as compared with conventional mice (TWA, *p* < 0.05). Non-classical monocytes were affected by pregnancy (TWA, *p* < 0.05); they were decreased in pregnancy in both conventional and germfree mice (TWA, followed by Fisher LSD test, *p* < 0.05). Non-classical monocytes were also increased by the germfree status (TWA, *p* < 0.05).

### The Expression of Activation Markers on Monocytes of Pregnant Mice

CD80 is a costimulatory molecule, which is upregulated on antigen presenting cells during activation. For both classical and intermediate monocytes, there was an interaction between pregnancy and germfree status (TWA, *P* < 0.05) (Figure 8B). Post testing showed a significantly increased expression of CD80 in pregnant vs. non-pregnant mice in both classical and intermediate monocytes, only in conventional mice (Fisher LSD test, *p* < 0.05), not in germfree mice. No effect of pregnancy or the microbiota status was found on CD80 expression in non-classical monocytes. (TWA, *p* < 0.05). MHCII expression of monocyte subsets was also affected by pregnancy (Figure 8C). MHCII in classical monocytes was decreased by pregnancy, both in conventional and germfree mice (TWA, followed by Fisher LSD test, *p* < 0.05) (Figure 8C). MHCII expression of intermediate monocytes was affected by pregnancy and the germfree status (TWA, *P* < 0.05). Only in conventional mice, not in germfree mice, MHCII expression was significantly decreased as compared with non-pregnant mice. There was no effect of either pregnancy or the germfree status on MHCII expression on non-classical monocytes.

### Correlation Between Microbiota and Immune Cells in Conventional Mice

**Figure 9:**
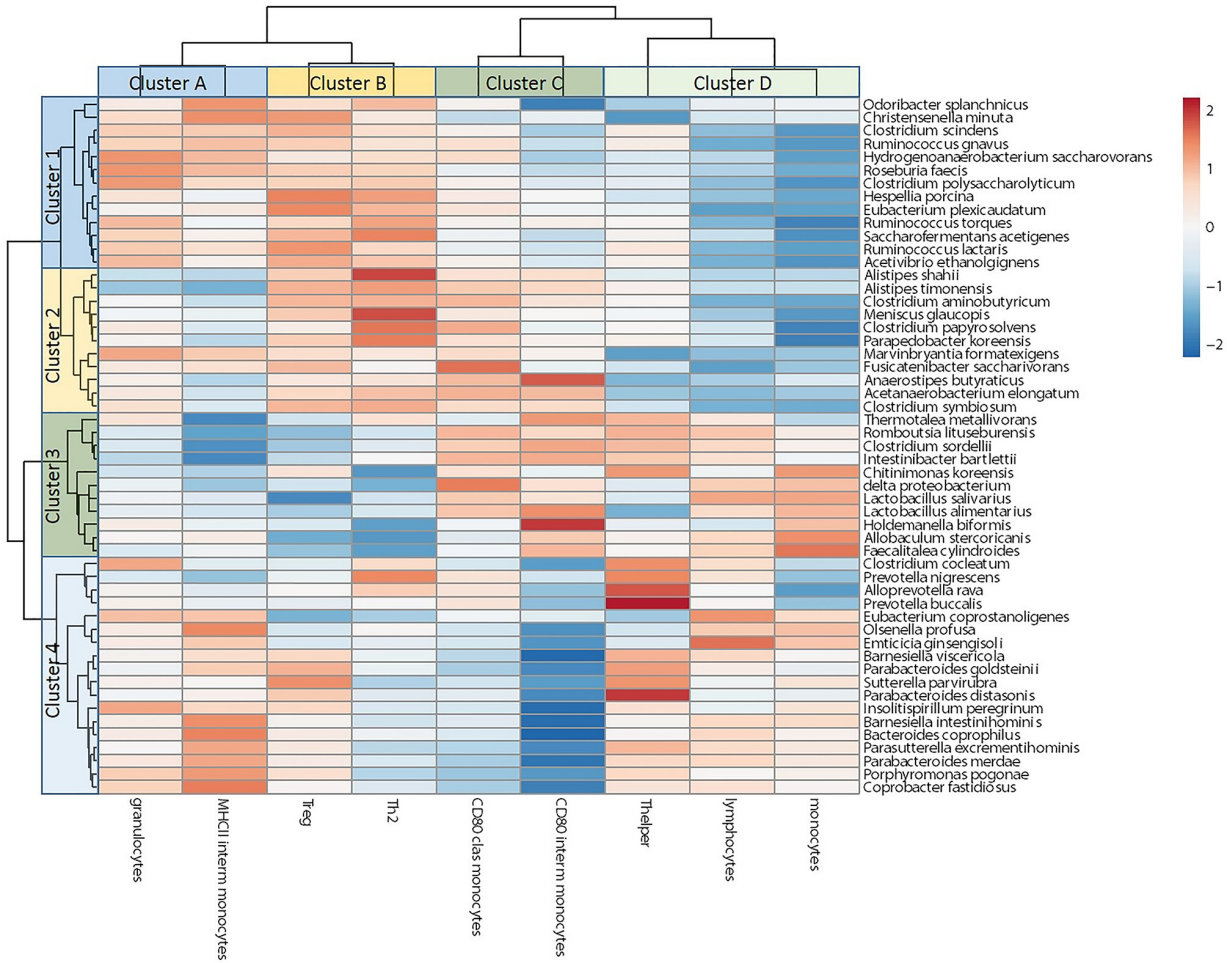
Correlation between microbiota species and immune cell populations in conventional pregnant mice. The figure shows a heatmap of Spearman's correlations coefficients after individual correlation of significantly different microbiota species between day 18 of pregnancy and pre-pregnancy (clusters 1–4) and immune cell populations with different adaptations in conventional vs. germfree mice (clusters A–D).

To gain insight into the relationship between the gut microbiota and immune cell changes during pregnancy in conventional mice, we correlated individual microbiota abundance data with immune cell data of the same mice. We used data from conventional day 18 mice: we used percentages of immune cells and percentage of immune cells expressing activation markers that were differently regulated by pregnancy in conventional vs. germfree mice. For microbiota data we used bacterial species from conventional mice of which the abundance was different at day 18 of pregnancy as compared with pre-pregnancy. Pearson's correlation coefficients are shown in a heatmap (Figure 9). Bacteria in cluster 1, of which most bacteria were upregulated during pregnancy, seem to be positively correlated with immune cell cluster A and B, which contains percentages blood granulocytes and percentages intermediate monocytes expressing MHCII (cluster A) and percentages of splenic FoxP3+ (Treg) and GATA3+ (Th2) cells (cluster B). Bacterial cluster 1 is negatively correlated with immune cell cluster D, which contains splenic percentages of Th cells and percentages of blood monocytes and lymphocytes. Bacterial cluster 2, which contains bacteria that are either upregulated or downregulated during pregnancy, was also negatively correlated to immune cells in cluster D and positively correlated to immune cells in cluster B. Bacteria in cluster 4 are negatively correlated with immune cells in cluster C, most strongly with percentage of CD80 expressing intermediate monocytes. Abundances of all of the bacteria in cluster 4 are significantly decreased during pregnancy.

## Discussion

In the present study we showed that during syngeneic pregnancy in mice, the composition of the gut microbiota changes. This composition has significantly changed at day 18 of pregnancy as compared with pre-pregnancy, with for instance an increased Firmicutes/Bacteroidetes ratio and a decreased Shannon index. Some bacterial changes can already be observed at day 14, for instance a significantly decreased abundance of *B. intestinihominis*. We also showed that in syngeneic conventional pregnant mice, immune responses change during pregnancy. We observed a decreased percentage of Th1 cells in pregnant conventional mice, as well as a trend toward an increased percentage of Th2 cells and an increased percentage of Treg cells. Monocyte subsets also changed in conventional syngeneic pregnant mice, with an increased percentage of classical monocytes and a decreased percentage of non-classical monocytes. Moreover, classical and intermediate monocytes showed increased expression of CD80 and decreased expression of MHCII during pregnancy in these mice. Next, we showed that the presence of microbiota may be important in some of these adaptations of the maternal immune response to pregnancy, since we found different adaptations in immune cell numbers of pregnant germfree mice compared with pregnant conventional mice, i.e., the increase in the splenic percentage of FoxP3+ lymphocytes and the trend toward an increased Th2 percentage was only observed in pregnant conventional mice, not in pregnant germfree mice. Moreover, increased CD80 expression on monocytes was only observed in conventional pregnant mice and not in germfree pregnant mice, while decreased MHCII expression was also only observed in conventional pregnant mice and not in germfree pregnant mice. Finally, we correlated the gut bacteria of conventional mice that were significantly changed at day 18 of pregnancy with the immune cells that were significantly changed in pregnant compared with non-pregnant mice in conventional pregnant mice only and showed that clusters of bacteria correlated with clusters of immune cells. Together these data indicate that the maternal gut microbiota may be involved in inducing some of the immunological adaptations to pregnancy.

We found various significant differences in phyla and species abundance at day 18 of pregnancy, with an increased abundance of Firmicutes and a decreased abundance of Bacteriodetes, Actinobacteria, Cyanobacteria, and Proteobacteria as compared with pre-pregnancy. At the species level, we found an increased abundance of for instance *A. stercoricanis, F. cylindroides, R. faecis and E. plexicaudatum, Holdemanella biformis*. These are all butyrate producing Firmicutes (32). Butyrate is an short chain fatty acid, which has immunomodulatory and anti-inflammatory effects (33). Butyrate can for instance promote differentiation and proliferation of Treg (34). We found decreased abundance of species like *B. Intestinihominis and B viscericola, P. pogonae, A. rava, O. splanchnicus* as well as 2 *Alistipes* species. Most of these bacteria are propionate producers (32). Propionate has been shown to have similar effects as butyrate on the immune response, f.i. in increasing Tregs, however, propionate has been shown to be less effective than butyrate (35). During syngeneic pregnancy in the mouse, there may be an increase in butyrate and a decrease in propionate production by the gut microbiota, which may result in increased plasma butyrate and decrease plasma propionate and this may affect the maternal immune response. This hypothesis needs to be tested in future studies.

The group of Koren et al. (27) was the first to show changes in the gut microbiota during pregnancy in humans. They, however, found an increase in Proteobacteria and Actinobacteria. Since our study have been done in syngeneic pregnancy, differences between our mouse study and the human study may be explained by the presence of the semiallogeneic fetus in the human study, as compared to syngeneic fetuses in our mouse study. Future studies are needed to confirm this hypothesis. We only studied one mouse strain, with one diet and at one location. However, we are not the only group who found differences in gut microbiota during pregnancy. Also previous studies in mice have shown an effect of pregnancy on the abundance of gut microbiota species (14, 36, 37). However, each of these mouse studies found their own specific adaptations of the microbiota to pregnancy. All of these studies have been done in syngeneic pregnancies. Differences between the studies may be due to differences in diet or technical differences in 16S rRNA sequencing (38). Another factor may be genetic differences between the mouse strains, since Elderman et al. has previously shown that changes in the microbiota at day 18 were strain dependent (14). Although in this study various differences in microbiota species were found between pregnant and non-pregnant mice in the from the BALB/c strain, no significant differences in microbiota were found between pregnant and non-pregnant C57BL/6 mice. In the present study, we also used C57BL/6 mice, but a different sub strain (C57BL/6JOlaHsd), and we did find differences in gut microbiota at day 18 of pregnancy. The difference with our previous study may thus be the sub strain used, but also the use of a different diet and the fact that we did a longitudinal study, which may more easily pick up differences in microbiota. The mechanism changing the microbiota during pregnancy are unclear at this time. However, it has been suggested that pregnancy hormones, like progesterone, may affect the gut microbiota (36). Also, diet has been shown to affect the gut microbiota (37).

In view of the known effects of the microbiota on the immune system, we hypothesized that the microbiota during mouse pregnancy may be involved in inducing adaptations of the immune response to pregnancy. Therefore, we sacrificed conventional and germfree syngeneic pregnant (at the end of pregnancy) and non-pregnant conventional and germfree mice and studied their immune response. In line with our previous results and results from others, we found various adaptations in the adaptive immune cells in syngeneic pregnant conventional mice. We showed an increased percentage of FoxP3+ cells and a trend toward an increase in GATA3+ cells, as well as a decrease in the percentage of Tbet+ cells in the spleens of conventional pregnant mice as compared to non-pregnant mice. This is in line with various data showing that the Th1/Th2 balance is decreased in pregnancy (4, 39, 40). Also, the increase in FoxP3+ (Treg cells) is in line with previous data (14). We, for the first time show that also syngeneic germfree mice do adapt their immune response to pregnancy. We found, similar to conventional pregnant mice, a decreased percentage of Tbet+ (Th1 cells) cells in the spleens of pregnant germfree mice as compared with non-pregnant germfree mice. However, in contrast to conventional pregnancy mice, in germfree pregnant mice the splenic percentage of FoxP3+ cells and GATA3+ cells were not different compared with germfree non-pregnant mice. This suggests that the increased splenic percentages of FoxP3+ and (trend to) increased GATA3+ cells in conventional mice at the end of pregnancy may be induced by the gut microbiota. This suggestion is in line with various papers indicating an effect of several bacterial species on Treg cells and Th2 cells (24, 25). Further studies are needed to evaluate whether the microbiota may affect the immune response in semiallogeneic pregnant mice.

In germfree pregnant mice, we found a slightly, but significantly increased number of fetal resorptions and a slightly decreased fetal weight. The mechanism of this increased number of fetal resorptions remains unknown from the present study. Various studies in the 1980s have shown that the housing environment of mice is associated with different numbers of fetal resorptions. Hamilton et al. showed that a cleaner environment (i.e., a specific pathogen free environment vs. a conventional environment) is associated with decreased numbers of resorptions in allogeneic pregnancies (CBA × DBA/2) (41), although other studies found the opposite effect (42) or no effect of these different environments (43). In all these studies, differences were only observed in semiallogeneic pregnancies and not in syngeneic pregnancies, suggesting that the mechanisms are related to rejection of the semiallogeneic fetus. The mechanism of increased fetal resorptions in germfree vs. conventional mice in our study must therefore be different, since we used syngeneic pregnancies. Further studies are therefore necessary to evaluate mechanisms of the increased fetal resorption in germfree mice. We do expect a role of the immune response, especially the immune response in the placenta and mesometrial triangle. Immune cells in the mesometrial triangle are very important for normal placentation. Differences in immune cells in the mesometrial triangle, such as decreased numbers of natural killer cells, may result in defective placentation, and therefore in fetal growth restriction or resorptions (44). We are evaluating the immune cells in the mesometrial triangle in conventional vs. germfree pregnant mice.

Individual mouse data from pregnant conventional mice from blood immune cells and splenic immune cells that were differently affected by pregnancy in germfree vs. conventional mice and individual microbiota species that were different between pregnant and non-pregnant conventional mice were correlated and clustered. The microbiota clustered in 4 clusters. Cluster 1 contained mainly bacteria that were upregulated during pregnancy, such as *R. faecis*, 2 *Ruminococcus* species and 3 *Clostridium* species. These bacteria positively correlated with percentage of Treg and Th2 cells (cluster B), suggesting that the species in this cluster may upregulate these immune cells. *Roseburia, Rumminococcus* and various commensal *Clostridium* species are known to produce short chain fatty acids (32, 45, 46), which in their turn are known to induce Tregs (47). Various bacterial species of clusters 1, 2, and 3 also correlated positively or negatively with immune cluster D, mainly with monocytes and lymphocytes. This suggests that the microbiota may be involved in hematopoiesis in pregnancy. Indeed, percentages of lymphocytes, monocytes and granulocytes are differently affected by pregnancy in conventional and germfree mice. The effect of the microbiota on hematopoiesis outside pregnancy has been shown before by Josefsdottir et al. (48). Percentages of blood monocyte subsets are affected by pregnancy, with an increase in the percentage of classical monocytes and a decrease in the percentage of non-classical monocytes during pregnancy in both conventional and germfree mice. Since classical monocytes mature in the circulation to non-classical monocytes (49), these data may suggest a decreased maturation of monocytes during pregnancy in these syngeneic mice. Since changes in monocyte subsets are similar in conventional and germfree mice, the microbiota is probably not involved and other pregnancy factors, such as cytokines or exosomes, may be involved in inducing these changes in monocyte subsets.

Bacterial cluster 4 contains only bacteria that were decreased during pregnancy. They were strongly negatively correlated with the expression of CD80 on intermediate monocytes and with the expression of CD80 on classical monocytes. Expression of CD80, which is an activation marker, on these monocyte subsets increased during conventional mouse pregnancy, suggesting that the bacteria in this cluster may normally be involved in downregulating CD80 expression, but due to their decrease in pregnancy, intermediate, and classical monocytes may upregulate CD80 expression. This cluster contains 2 *Barnesiella* species, which have been shown to be anti-inflammatory in mice (50). This suggestion of downregulation of CD80 expression on monocytes by certain bacterial species is in line with the fact that in non-pregnant germfree mice, CD80 expression on intermediate monocytes is increased vs. non-pregnant conventional mice (Figure 8B). A similar reasoning would apply to MHC II expression on intermediate monocytes, which is down regulated during pregnancy in conventional mice and positively correlated with the decreased bacteria in cluster 4 suggesting that normally these bacteria upregulate MHC II, but when these bacteria are decreased during pregnancy, MHC II expression is decreased.

In conclusion, our data, together with previous data in mice and humans, indicate that the gut microbiota change during pregnancy. The changes, however, may be species and strain dependent, but may also depend on the diet as well as on the method of 16S RNA sequencing. In the present study, we found an increase in Firmicutes species (such as *A. stercoricanis*) and a decrease in Bacteroidetes species (such as *B. intestihominis, B. viscericola*). The gut microbiota in pregnant mice may be involved in some of the adaptations of the maternal immune response to pregnancy, especially in the increase in Treg and the increased activation of monocytes at the end of pregnancy. Although these data show that the microbiota may be involved in inducing adaptations of the immune response to pregnancy, these data do not show whether the change observed in the microbiota during pregnancy are responsible for these immunological adaptations. However, the fact that we found various correlations between the pregnancy-induced adaptations in immune cells and the pregnancy-induced changes in bacterial species in conventional mice, may suggest a role for the changes in the microbiota at day 18 of pregnancy in the adaptations of the immune response. Further studies, such as studying the immune response after transplantations of fecal material from pregnant and non-pregnant conventional mice into pregnant and non-pregnant germfree mice, are needed to confirm this hypothesis.

## Data Availability Statement

The raw data supporting the conclusions of this manuscript will be made available by the authors, without undue reservation, to any qualified researcher.

## Ethics Statement

The animal study was reviewed and approved by the Central Committee on Animal Testing, Netherlands.

## Author Contributions

The experiments were designed and setup by MF and PV. YL and TB performed experiments. MF, CL-B, HH, and PV wrote the manuscript.

### Conflict of Interest

CL-B was employed by the company Yili Innovation Center Europe B.V., Wageningen, The Netherlands. The remaining authors declare that the research was conducted in the absence of any commercial or financial relationships that could be construed as a potential conflict of interest.
