# Supplementary material for: Microbiota Induced Changes in the Immune Response in Pregnant Mice
Source: Front Immunol. 2020 Jan 9;10:2976. doi: 10.3389/fimmu.2019.02976 (PMC6962187; doi:10.3389/fimmu.2019.02976)
Supplement: Supplementary file 1 [file Data_Sheet_1.PDF]

species	Mean abundance pre-pregnancy	Mean abundance day 7	Mean abundancy day 14	Mean abundance day 18	Sign day 7	Sign day 14	Sign day 18
Allobaculum stercoricanis	35.06	32.11	37.44	52.82	ns	ns	*
Barnesiella intestinihominis	18.43	18.21	12.47	4.79	ns	*	*
Porphyromonas pogonae	6.03	6.40	6.68	3.19	ns	ns	*
Barnesiella viscericola	4.27	4.36	3.34	1.56	ns	ns	*
Faecalitalea cylindroides	2.70	2.36	2.70	3.84	ns	ns	*
Clostridium leptum	1.87	2.02	2.56	2.95	ns	ns	ns
Olsenella profusa	2.33	2.16	1.98	1.27	ns	ns	*
Clostridium papyrosolvens	1.44	1.49	0.46	0.30	ns	*	*
Sutterella parvirubra	1.21	1.47	2.20	0.69	ns	ns	*
Parasutterella excrementihominis	1.74	1.56	1.53	1.01	ns	ns	*
Bifidobacterium animalis	1.16	1.03	1.26	1.14	ns	ns	ns
Alistipes finegoldii	0.92	2.27	1.56	0.85	*	ns	ns
Acetatifactor muris	0.97	1.34	1.05	1.05	ns	ns	ns
Lactobacillus johnsonii	1.30	1.14	1.18	0.84	ns	ns	ns
Clostridium fusiformis	0.60	0.81	0.95	1.34	ns	ns	ns
Clostridium jejuense	0.37	0.62	0.75	0.39	ns	ns	ns
Blautia coccoides	0.59	0.73	0.80	1.09	ns	ns	ns
Flavonifractor plautii	0.61	0.58	0.81	1.29	ns	ns	ns
Eisenbergiella tayi	0.51	0.71	0.87	1.20	ns	ns	ns
Alistipes senegalensis	0.56	0.58	0.95	0.68	ns	ns	ns
Olsenella umbonata	0.89	0.69	0.74	0.41	ns	ns	ns
Romboutsia ilealis	0.54	0.59	1.00	1.18	ns	ns	ns
Alloprevotella rava	0.96	0.92	0.70	0.20	ns	ns	*
Desulfovibrio desulfuricans	0.55	0.59	0.69	0.66	ns	ns	ns
Natronoflexus pectinivorans	0.52	0.66	0.72	0.41	ns	ns	ns
Akkermansia muciniphila	0.39	0.69	0.34	0.14	ns	ns	ns
Bifidobacterium pseudolongum	0.81	0.52	0.52	0.52	ns	ns	ns
Alistipes putredinis	0.14	0.32	0.27	0.26	ns	ns	*
Bacteroides acidifaciens	0.39	0.47	0.36	0.42	ns	ns	ns
Lactobacillus murinus	0.41	0.35	0.50	0.27	ns	ns	ns
Mucispirillum schaedleri	0.14	0.30	0.40	0.71	ns	ns	ns
Roseburia faecis	0.23	0.24	0.40	0.53	ns	ns	*
Anaerotruncus colihominis	0.28	0.33	0.42	0.51	ns	ns	ns
Lysinibacillus xylanilyticus	0.27	0.24	0.19	0.28	ns	ns	ns
Lactobacillus vaginalis	0.39	0.34	0.54	0.32	ns	ns	ns
Bacteroides vulgatus	0.48	0.55	0.35	0.10	ns	ns	ns
Dorea longicatena	0.36	0.41	0.40	0.24	ns	ns	ns
Butyrivibrio crossotus	0.30	0.21	0.57	0.29	ns	ns	ns
Clostridium viride	0.25	0.27	0.29	0.25	ns	ns	ns
Odoribacter splanchnicus	0.37	0.30	0.21	0.14	ns	*	*

Lactobacillus salivarius	0.21	0.20	0.27	0.39	ns	ns	*
Parabacteroides distasonis	0.21	0.27	0.18	0.08	ns	ns	*
Parabacteroides goldsteinii	0.24	0.23	0.20	0.13	ns	ns	*
Clostridium propionicum	0.19	0.24	0.25	0.19	ns	ns	ns
Clostridium polysaccharolyticum	0.12	0.18	0.20	0.32	ns	ns	*
Parabacteroides merdae	0.24	0.21	0.16	0.10	ns	ns	*
Bilophila wadsworthia	0.17	0.17	0.28	0.30	ns	ns	ns
Clostridium aminobutyricum	0.23	0.23	0.16	0.08	ns	ns	*
Alkaliphilus transvaalensis	0.12	0.11	0.13	0.19	ns	ns	ns
Hespellia porcina	0.09	0.13	0.20	0.24	ns	ns	*
Clostridium populeti	0.19	0.16	0.14	0.17	ns	ns	ns
Christensenella minuta	0.13	0.11	0.09	0.07	ns	ns	*
Enterorhabdus mucosicola	0.18	0.16	0.20	0.20	ns	ns	ns
Odoribacter laneus	0.17	0.17	0.10	0.13	ns	ns	ns
Ruminococcus lactaris	0.08	0.12	0.17	0.25	ns	ns	*
Ruminococcus gnavus	0.20	0.09	0.08	0.16	ns	ns	ns
Alistipes shahii	0.14	0.24	0.17	0.06	ns	ns	*
candidate division	0.15	0.11	0.12	0.15	ns	ns	ns
Parasutterella secunda	0.11	0.12	0.20	0.10	ns	ns	ns
Prevotella buccalis	0.22	0.22	0.14	0.05	ns	ns	*
Clostridium methylpentosum	0.09	0.10	0.15	0.21	ns	ns	ns
Clostridium lactatifermentans	0.09	0.12	0.12	0.16	ns	ns	ns
[Clostridium] scindens	0.07	0.09	0.11	0.22	ns	ns	*
delta proteobacterium	0.13	0.16	0.06	0.01	ns	ns	*
Parvibacter caecicola	0.14	0.18	0.14	0.13	ns	ns	ns
Eubacterium plexicaudatum	0.04	0.09	0.12	0.32	ns	ns	*
[Clostridium] sporosphaeroides	0.10	0.08	0.12	0.20	ns	ns	ns
Marvinbryantia formatexigens	0.06	0.09	0.11	0.19	ns	ns	t
Hydrogenoanaerobacterium saccharovorans	0.06	0.09	0.14	0.20	ns	ns	*
Clostridium saccharolyticum	0.07	0.08	0.07	0.04	ns	ns	ns
Prevotella nigrescens	0.14	0.14	0.12	0.04	ns	ns	*
Collinsella tanakaei	0.12	0.11	0.10	0.11	ns	ns	ns
Acetivibrio ethanolgignens	0.06	0.07	0.10	0.14	ns	ns	*
Mucinivorans hirudinis	0.08	0.10	0.07	0.07	ns	ns	ns
Eubacterium coprostanoligenes	0.12	0.10	0.03	0.01	ns	ns	*
Olsenella uli	0.09	0.07	0.08	0.11	ns	ns	ns
[Clostridium] aldenense	0.07	0.04	0.06	0.10	ns	ns	ns
Stomatobaculum longum	0.06	0.07	0.06	0.11	ns	ns	ns
Eubacterium ramulus	0.07	0.07	0.11	0.12	ns	ns	ns

Dorea formicigenerans	0.07	0.08	0.08	0.11	ns	ns	ns
Parabacteroides chartae	0.09	0.09	0.10	0.06	ns	ns	ns
Meniscus glaucopis	0.13	0.09	0.05	0.03	ns	ns	*
Sphingobacterium mizutaii	0.08	0.09	0.06	0.05	ns	ns	ns
Insolitispirillum peregrinum	0.13	0.12	0.04	0.01	ns	ns	*
Faecalibacterium prausnitzii	0.07	0.05	0.08	0.03	ns	ns	ns
Intestinimonas butyriciproducens	0.06	0.07	0.07	0.05	ns	ns	ns
Clostridium hylemonae	0.05	0.05	0.05	0.09	ns	ns	ns
Alistipes timonensis	0.09	0.08	0.07	0.03	ns	ns	*
[Clostridium] sordellii	0.03	0.04	0.07	0.16	ns	ns	*
Coprobacter fastidiosus	0.08	0.08	0.06	0.03	ns	ns	*
Acetanaerobacterium elongatum	0.03	0.05	0.07	0.09	ns	ns	*
Roseburia inulinivorans	0.06	0.05	0.03	0.04	ns	ns	ns
Thermotalea metallivorans	0.04	0.05	0.04	0.08	ns	ns	*
Robinsoniella peoriensis	0.04	0.05	0.04	0.08	ns	ns	ns
Prevotella ruminicola	0.08	0.06	0.06	0.03	ns	ns	ns
Anaerostipes butyraticus	0.03	0.03	0.03	0.10	ns	ns	*
Lactobacillus alimentarius	0.03	0.03	0.04	0.07	ns	ns	*
Tyzzerella nexilis	0.05	0.05	0.04	0.07	ns	ns	ns
Pseudoflavonifractor capillosus	0.04	0.05	0.04	0.06	ns	ns	ns
Clostridium celerecrescens	0.04	0.03	0.05	0.05	ns	ns	ns
Saccharofermentans acetigenes	0.05	0.02	0.01	0.01	ns	*	*
Dehalobacterium formicoaceticum	0.05	0.06	0.04	0.03	ns	ns	ns
Anaeroplasma bactoclasticum	0.02	0.03	0.03	0.04	ns	ns	ns
Holdemanella biformis	0.04	0.03	0.04	0.06	ns	ns	*
Emticicia ginsengisoli	0.06	0.04	0.04	0.02	ns	ns	*
Romboutsia lituseburensis	0.03	0.03	0.07	0.08	ns	ns	*
Intestinibacter bartlettii	0.01	0.02	0.04	0.13	ns	ns	*
Chitinimonas koreensis	0.03	0.05	0.06	0.02	ns	ns	*
Parapedobacter koreensis	0.05	0.04	0.03	0.03	ns	ns	*
[Clostridium] cocleatum	0.05	0.03	0.03	0.00	ns	ns	t
Porphyromonas canis	0.04	0.05	0.06	0.04	ns	ns	ns
[Clostridium] symbiosum	0.02	0.03	0.03	0.08	ns	ns	*
Fusicatenibacter saccharivorans	0.01	0.02	0.05	0.11	ns	*	*

Supplementary table 1: Abundance of bacterial species pre-pregnancy and at various day of pregnancy.

*: Significantly different from pre-pregnancy; Friedman's test followed by Dunn's test, $p<0.05$ ns= not significant from prepregnancy
